# At the Royal Westminster Ophthalmic Hospital

**Published:** 1889-07-20

**Authors:** 


### The Hospital Workshop.

### No. VI.—AT THE ROYAL WESTMINSTER OPHTHALMIC HOSPITAL.

(BY OUR SPECIAL COMMISSIONER.)

This is a building of the old-fashioned sort, founded in 1816, before the days of plate-glass and æsthetic furnishings. Miss Rouse, the present matron, is doing a great deal to brighten up the place, and your commissioner was given to understand that a thorough overhauling of the internal arrangements is in contemplation. The wards have room for 50 beds, and 334 persons were admitted during the year 1888. By far the greater number, however, are treated in the out-patient department, and 7,947 cases, with an average of 70,000 attendances, are reported for the same twelve months. Upon arrival applicants go to the waiting-room, whence they pass in batches to a large hall, where the surgeons for the day are on duty. After consultation each patient takes from the table a numbered ticket, which settles the order of obtaining medicine at the dispensary. This simple plan works smoothly enough in practice.

The Westminster Ophthalmic Hospital claims to have its doors open to the poor day and night. Treatment here is entirely free, and not, as stated by the Charity Organisation Society in its late memorandum, "free, but patients are expected to pay for their maintenance." The secretary, Mr. Beattie Campbell, estimates that three-fourths of those relieved belong to the really poor, while the others are made up of clerks, governesses, warehousemen, and the like. All particulars of applicants are registered and verbal enquiries made by a clerk. Cases found unsuitable—perhaps one a week—are sent away or advised to go to consultants. This test may seem unsatisfactory to those who demand a rigid investigation into the means of persons seeking hospital relief. But the general practitioners do not complain so much of hospitals where a highly specialised knowledge is required. Still, even in eye-work, there are many chronic cases that might safely be handed over to their own medical men, to whom they would pay moderate fees. Such a course could hardly fail to economise hospital funds, and, at the same time, the poor, the public, and the rank and file of a struggling profession would be more fairly dealt with.

Patients flock to the "Westminster" from all parts of London, but chiefly, perhaps, from Battersea, Chelsea, and the West End generally. Inmates of the workhouses are sent here, and occasionally some of Dr. Barnardo's waifs and strays. Beyond these, many hail from a distance, chiefly for operation, or from foreign parts, such as Australia, New Zealand, British Guiana, India, and British Honduras.

A glance into the out-patient room shows a crowd of persons of all ages. Many of them wear bandages or eye-shades, and are patiently waiting their turn while others are being examined by the surgeons. Of the eyes, a few look clear and bright, a number are squinting and blinking, and here and there is the unmistakable look of blindness. There are dark rooms, where the interior of the eyeball is searched by means of the "ophthalmoscope"—a small mirror with a hole in the centre, through which the observer looks, and which is now often lighted up by electricity. Hanging from the walls are "test types"—printed letters of different sizes—before which patients are being tried with spectacle frames, fitted with lenses from the large boxes that lie close at hand. There is much more of interest, but leaving all these things, let us take a stroll through the homely and comfortable wards.

Here is a baby of fifteen months. Three weeks ago an attack of measles set in, and the destruction of one eye has followed so rapidly that its removal is now necessary, and the mother is waiting with the child in her arms until it is time to go into the operating theatre. The poor woman comes from Marylebone, and does "washing and charing." Her husband is a house-painter's labourer, and at home there are four more children, but they have all had measles.

Standing near is a girl of fourteen who comes from Bagshot Heath, which she reminds us is twenty-seven miles away. She has been suffering from a painless loss of sight for the last two years. It came on gradually, and the "cornea," as the clear front wall of the eye is called, is dull on each side like ground glass. Under treatment these cases sometimes regain vision in a most wonderful manner, and this patient has improved so much since admission that there is every hope of her getting back a fair amount of sight.

The removable causes of blindness are few. Sometimes a clouded cornea may be cleared up, as in the foregoing case, or when there is a spot in the line of vision, a piece of the "iris," or coloured part round the "apple," may be cut away, making, as it were, a new pupil. When the lens or pupil is opaque it is called cataract, the chief varieties being "hard" and "soft." The operation for the hard kind consists in cutting through the upper half of the cornea, taking away a piece of the iris, and gently pressing out the lens. By far the greater number of cases of restored sight are due to this procedure. The patient is not operated on so long as he can see to do his work, as until then the cataract is not "ripe." About one-third of the total number of operations mentioned in the report are for cataract, and failures do not, as a rule, exceed five out of a hundred cases so treated.

A cheerful-looking old dame of sixty-eight is sitting in an armchair. She has the stamp of a country woman, and tells us on enquiry that her name is Mitten, and she was brought up in a Northamptonshire village, although for many years she has lived at Clapham. Two years ago her sight began to fail, and four months back she came to this hospital to have her right eye operated upon. With the help of glasses she can now see to read large print, to knit, and to recognise her friends. The case is one of double cataract, and the poor old lady is waiting for Mr. Adams Frost to operate on the other eye, let us hope with a like success that attended his treatment of the first.

A great many children are admitted with accidents to their eyes. Here is a girl of nine from Pimlico. Ten days ago she struck her eye with the point of a large wooden meat skewer while playing at tip-cat. Although she suffered a great deal of pain at the time, the cornea was not wounded, but bleeding took place into what is known as the "arterior chamber" of the eyeball. The reader may imagine the appearance of this red mass—not unlike a polished carbuncle—hiding the pupil and iris, and entirely cutting off vision on that side. Treatment consisted in putting the girl to bed with a bandage over her eyes. Now the blood is being rapidly absorbed, so that she will soon be quite right again and able to return home.

Another child who has met with an accident. This time a very small boy who rejoices in the somewhat remarkable name of Oscar Hulin, and we gather from the card hanging over his bed that his employment is "at school." Eight days ago he was playing with another boy who smashed a ginger-beer bottle on the ground. Oscar then broke one of the pieces and a fragment flew up and struck him in the eye. One corner of the bandage round his head is lifted up, so that he can see with his sound eye the toys lying on his bed. At first he is rather shy, but soon grows friendly, and volunteers the information "My dada's a policeman," adding a little later, "he is going to lock that boy up." "Did it hurt you very much, Oscar?" "Yes, it dud." "Was it a soda-water bottle?" "Yes, it wus." "Did the other boy take the cork out?" "Yes, he dud." A little later, however, he remarked "the cork worn't in the bottle." Then his evidence became still more contradictory as to who broke the piece that cut his eye, but he always came back to the comforting remembrance of his father's official position. The notes of the case stated that a piece of the iris protruded through the severed cornea, and there was also a large cut on the nose. Under chloroform the projecting iris was removed, and the wounds stitched up. Most likely the eye will recover, but the pupil will be irregular, instead of round, and he will have to wear spectacles.

A young man of twenty-six, who five years ago had his left eyeball removed after injury from a splinter of iron. His trade was hammerman to a wheelwright, an employment which he further described as "hammering and vicing and drilling." Failing that occupation, he has taken to platelaying or any other makeshift that offered itself. Two years ago the remaining eye became bad—as commonly happens after removal of one globe. Although his sight has somewhat improved under good feeding and general treatment, his prospects of ultimate recovery are not promising.

Of late years great advances have been made in the study of the eye. For instance, in testing errors of vision—long, short, or irregular sight—much improvement has been made on the old-fashioned plan. Formerly different lenses were tried in a spectacle frame until the patient could read a certain-sized print at a fixed distance. The new process, "retinoscopy," consists in flashing the light of a mirror across the eyeball, and the surgeon can get accurate results by this method without asking a single question. People who trust the care of their sight to any but specialists skilled in this branch of work, are certainly not giving their eyes the benefit of the most advanced and scientific treatment.

The last case that will be mentioned is that of a boy of seven, who, instead of having his eyeball removed, has undergone the new operation introduced by Mr. Mule, of Manchester. The old method would have been to take away the globe altogether, cutting through the muscles and optic nerve as far back as possible. In some cases resulting inflammation has reached the brain, and this suggested to Mr. Mule the need of a new operation, which, after many trials, is now nearly perfect. Your commissioner, on a previous occasion had the privilege of seeing the operation performed by Mr. Adams Frost, assisted by Mr. Sydney Stephenson. It consists briefly in scooping out the contents of the eyeball, and introducing a hollow glass ball, which is sewn up and allowed to remain there permanently. Antiseptic surgery has alone made such a result possible. This system has many advantages. The eye muscles not being cut, a movable stump is left to which the artificial eye may be fixed. But another most important and unlooked-for result is that under this method the sight of the remaining eye is not affected, as almost always happened, sooner or later, under the old method. The eyeball is so wonderfully protected within its bony case that the patient need feel no fear about carrying a glass ball in that position. It should be added there is a flourishing school attached to the hospital.

